# Changes in Visual Field Indices after Pterygium Surgery

**DOI:** 10.14744/bej.2021.21939

**Published:** 2021-12-17

**Authors:** Fikret Ucar, Halil Ibrahim Yener, Servet Cetinkaya, Huseyina Ture

**Affiliations:** 1. Department of Ophthalmology, Konya Eye Hospital, Konya, Turkey; 2. Department of Ophthalmology, Selcuklu Hospital, Karaman, Turkey

**Keywords:** Mean deviation, pattern standard deviation, pterygium surgery, visual field index, visual field tests

## Abstract

**Objectives:**

This study was a prospective evaluation of changes in the results of visual field tests taken before and after pterygium excision.

**Methods:**

This was a prospective, single-center study. Seventy-five eyes of 75 patients who had undergone pterygium excision with autograft implantation were enrolled. All of the patients had stage III pterygium according to the Johnston classification. The mean deviation (MD), pattern standard deviation (PSD), and visual field index (VFI) global index changes after pterygium excision were compared to evaluate the effect of pterygium on visual field analysis.

**Results:**

The mean preoperative MD value was -3.04±2.63 dB (range: -14.84–0.62 dB) and the mean postoperative MD value was -1.83±2.09 dB (range: -13.82–1.74 dB) (p<0.001). The mean preoperative PSD value was 2.59±1.92 dB (range: 1.16–12.76 dB) and the mean postoperative PSD value was 2.41±1.62 dB (1.15–13.29 dB) (p>0.05). The mean preoperative VFI value was 96.01±4.46% (range: 68–100%) and the mean postoperative VFI value was 96.28±4.18% (range: 70–100%) (p>0.05).

**Conclusion:**

After pterygium excision, the MD improved significantly. However, the PSD and VFI did not change significantly. The significant change in MD value was related to the reduction in corneal light scattering, contrast sensitivity, aberrations, and blockage on the optic axis.

## Introduction

Pterygium is the wing-shaped growth of conjunctival tissue, which extends from the limbus to the cornea. It is composed of hyperplastic and denatured collagen tissue with elastotic degeneration (1) and has a fibrovascular structure. It may be located on the nasal or temporal cornea. Ultraviolet light contributes considerably to the formation of pterygium (2). Pterygium is more commonly seen in males (3). It is generally more common between 20 and 40 years of age (4). In 20 meta-analytic studies, the prevalence of pterygium is placed variously in the range of 6.3–16.1% (10.2%) (5). Pterygium causes abnormalities in the tear film, induces astigmatism, photophobia, epiphora, blurred vision, and binocular diplopia due to Tenon capsule contraction leading to restricted eye movements. Pterygium also causes irritation, foreign body sensation, and dryness (1).

Visual field analysis provides us important information in both diagnosis and follow-up of the diseases such as glaucoma and optic neuropathies. In recent years, the Swedish Interactive Threshold Algorithm (SITA) became popular (6, 7). As compared to previous SITA full-threshold visual field tests, this test needs less time to conduct and the quality of the resultant data is better (8). The global indices of visual field analysis are mean deviation (MD) and pattern standard deviation (PSD).

These indices are used to interpret the visual field test. MD is the mean difference between the patient’s sensitivity and his/her age-matched control value. PSD is the deviation of every test point according to the patient’s visual acuity and age, and it is expressed as a decibel (dB) value (9, 10). MD compares all test points with normal reference visual field and, thus, it shows depression or elevation of the whole area in comparison with the normal area. MD increase indicates generalized depression or partial visual field defect. PSD indicates the standard deviation of the difference between the expected and measured values at every test localization. It shows us how much the measured visual field analysis is different from the normal age-matched visual field analysis. A lower PSD points to a regular visual field profile, a higher PSD points to an irregular visual field profile (10-12).

Visual field index (VFI) uses a pattern deviation map to determine abnormal visual field test points. If MD is worse than −20 dB, it uses the total deviation map. VFI evaluates the sensitivity of all locations with central weighting which is based on the frequency of ganglion cells. During the follow-ups of disease progression, VFI is more important for central visual field evaluation than the peripheral visual field. VFI is expressed as a percentage. It ranges from 100% (perimetrically normal) to 0% (perimetrically blind) (9).

We think that pterygium may affect visual field indices due to the reasons such as the reduction in corneal light scattering, contrast sensitivity, and optical aberrations. To the best of our knowledge, there has not been any study evaluating the effect of pterygium on automatic perimetry and visual field indices. In this study, we evaluated changes in the results of visual field tests taken before and after pterygium excision.

## Methods

This is a prospective, single-center study. The protocol of the study was approved by the local ethics committee (KTO Karatay University Faculty of Medicine Ethics Committee, Konya, Turkey). An informed written consent form was obtained from all patients before the surgery. The study was carried out according to the tenets of the Declaration of Helsinki.

Seventy-five eyes of 75 patients who had undergone pterygium excision with autograft implantation between June 2020 and December 2020 were enrolled in this study. All patients had Stage III pterygium (nasal or temporal) according to Johnston classification (13) and were older than 18 years of age (Table 1). Then, exclusion criteria were the use of miotic drops, any ocular diseases which might affect the visual field test, such as glaucoma and optic neuropathy, and low SITA index (false-positive responses, false-negative responses, and/or fixation losses >15%).

**Table 1.** Johnston Pterygium Classification

Stage 0	Pingueculum, posterior to the Limbus.
Stage 1	Tissue involvement to the Limbus.
Stage 2	Tissue just on to the Limbus.
Stage 3	Tissue between the Limbus and pupillary margin.
Stage 4	Tissue to central to the pupillary margin.

All of the operations were performed by the same surgeon (FU). In the post-operative period, all patients were administered moxifloxacin/dexamethasone combination drops (Moxidexa, Abdi Ibrahim, Turkey) 5 times a day for 2 weeks. Visual field analysis (Humphrey Field Analyzer Model 640; Humphrey Instruments, Inc., San Leandro, CA, USA) with 24:2 SITA-Fast strategy program and size III white stimulation were applied to all patients 1 month before and 3 months after the surgery. All tests were performed by the same technician.

**Figure 1.**
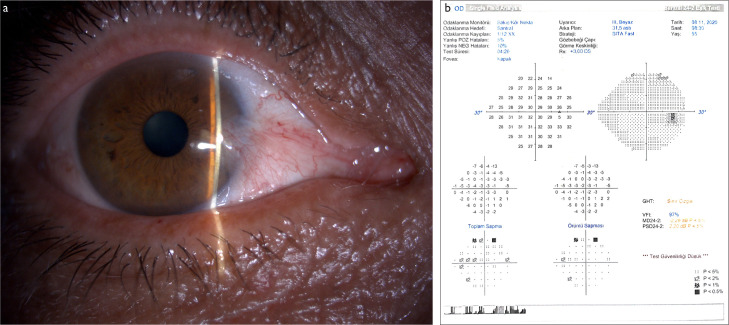
**(a)** Preoperative pterygium view. **(b)** Preoperative visual field test. Visual field indices; mean deviation (MD): -2.29 dB, pattern standard deviation (PSD): 2.20 dB, visual field index (VFI): 97%.

**Figure 2.**
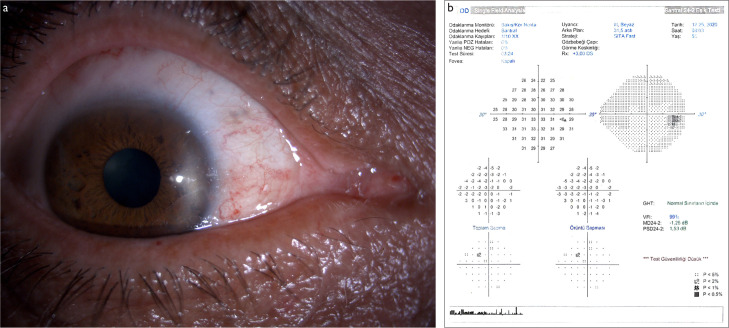
**(a)** Postoperative pterygium view. **(b)** Postoperative visual field test. Visual field indices; mean deviation (MD): -1.25 dB, pattern standard deviation (PSD): 1.53 dB, visual field index (VFI): 99%.

To evaluate the effect of pterygium on visual field analysis, we compared MD, PSD, and VFI global index changes after pterygium excision (Figs. 1a and b, 2a and b).

Statistical analysis was made using SPSS Version 22. To compare data, a paired t-test was used. P-value smaller than 0.05 was accepted as statistically significant.

## Results

**Figure 3.**
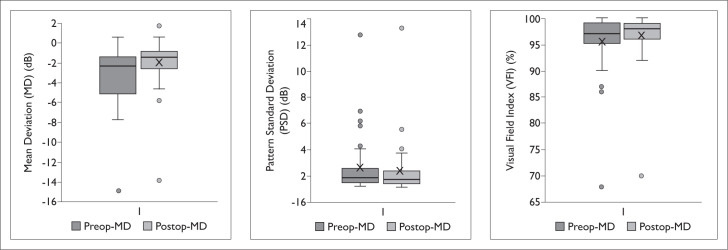
Preoperative and postoperative visual field indices changes.

Of the 75 patients, 39 patients (52%) were male, and 36 patients (48%) were female. The mean age of the patients was 48.69±14.44 (24–84) years of age. Visual field analyses were performed 1 month before and 3 months after the surgery. Pre-operative and post-operative MD, PSD, and VFI indices are presented in Table 2 and Figure 3. The mean pre-operative MD value was −3.04±2.63 (−14.84–0.62) dB and the mean post-operative MD value was −1.83±2.09 (−13.82–1.74) dB (p<0.001). The mean pre-operative PSD value was 2.59±1.92 (1.16–12.76) dB and the mean post-operative PSD value was 2.41±1.62 (1.15–13.29) dB (p>0.05). The mean pre-operative VFI value was 96.01±4.46 (68–100) percent and the mean post-operative VFI value was 96.28±4.18 (70–100) percent (p>0.05).

**Table 2.** The mean preoperative and postoperative MD, PSD and VFI values

**Parameters**	**Preoperative Average**	**Postoperative Average**	**p-value**
MD (dB)	-3.04±2.63 (-14.84)–(-0.62)	-1.83±2.09 (-13.82)–(-1.74)	0.00
PSD (dB)	2.59±1.92 (1.16)–(12.76)	2.41±1.62 (1.15)–(13.29)	0.13
VFI (%)	96.01±4.46 (68)–(100)	96.28±4.18 (70)–(100)	0.36

MD: Mean deviation; PSD: Pattern standard deviation; VFI: Visual field index; dB: Decibel.

## Discussion

Many studies reported visual field changes after cataract extraction operations on patients with cataract and glaucoma. Many studies reported that cataract extraction improved MD both in eyes with glaucoma and without glaucoma (14-17). Budenz et al. (18) simulated the effect of cataracts on glaucomatous visual fields by putting frosted glass filters on the patients’ eyes. They concluded that the light scattering effect of the cataract depressed glaucomatous area in every test location equally regardless of original threshold sensitivity. Pterygium, which is a peripheral corneal opacity, induces corneal light scattering which leads to background lightning of retina and decreases the contrast sensitivity (19). Lin et al. (20) reported that contrast sensitivity decreased, and glare disability occurs in patients with pterygium.

A few studies were aimed at establishing the relation of pterygium and its surgery with wavefront aberrations (21-25). Minami et al. (24) reported that as the diameter of pterygium increases, high-order aberrations of cornea increase, too. Ozgurhan et al. (25) stated that surgical removal of primary and recurrent pterygium significantly decreased high-order aberrations such as trefoil and coma. Pesudovs et al. (22) also stated that pterygium extraction decreased residual aberrations significantly.

PSD and VFI did not improve significantly after pterygium surgery. However, MD improved significantly. It is difficult to attribute these changes to the learning effect. The previous studies evaluating the learning effect of patients with glaucoma did not show any difference in PSD value (26). It has been reported that there may be an improvement in MD value associated with the learning effect of 0.6 dB in the right eye and 0.8 dB in the left eye (26). However, our results are quite different from the learning effect and it is not possible to relate these results to the learning effect.

We think that MD improvement after the operation is related to a decrease in light scattering, and a reduction in contrast sensitivity and aberrations. However, further investigations are needed to confirm this. The opacities inside the optic axis, such as lens opacity, cataract, corneal opacity, and pterygium may cause visual field changes (27-30). It was reported that, in 22 patients with pterygium, visual fields were affected by the size of the lesion, and in another patient, after pterygium excision, visual field defect disappeared (30).

Lam et al. (17) reported that after cataract extraction, MD decreased significantly, but PSD did not change. In our study also, PSD did not change significantly after pterygium excision.

In glaucoma patients, the presence of pterygium may affect visual field analysis and thus may further complicate the case. Therefore, pterygium excision is important in follow-ups of glaucoma patients. In our search for literature, we came across only one study related to the effect of pterygium on visual field analysis. However, the researchers had used manual perimetry. Ours is the first study in which visual field changes have been evaluated, before and after pterygium surgery, with automatic perimetry.

## Conclusion

After pterygium excision, MD improves significantly. However, PSD and VFI do not change significantly. The significant change in MD value is related to the reduction in corneal light scattering, contrast sensitivity, aberrations, and blockage on the optic axis.

### Disclosures

**Ethics Committee Approval:** KTO Karatay University Ethics Committee, protocol number: 2020/007, Date: 19/06/2021.

**Peer-review:** Externally peer-reviewed.

**Conflict of Interest:** None declared.

**Authorship Contributions:** Involved in design and conduct of the study (FU, HİY, SÇ); preparation and review of the study (FU, SÇ); data collection (FU); and statistical analysis (FU, HT).
